# 

**Published:** 1894-03-24

**Authors:** 


**Notice to Advertisers and Subscribers.**

All Advertisements, Orders for Copies of The Hospital, and Business Communications should be addressed to The Publisher, *not to the Editor*, The Hospital, 428, Strand, W.C.

The Cost of Subscription, post free, is as follows:—Per week, 2½d.; per quarter, 2s. 8d.; per half-year, 5s. 3d.; per annum, 10s. 6d. Foreign:—Per Annum, 12s. 8d.; payable, in all cases, in advance.

**OUR NEW OFFICES.**

428, Strand, W.C., will henceforth be the Office of this Journal.

**Notices of Births, Deaths, and Marriages** are inserted in this column at a charge of 2s. 6d. for an announcement not exceeding four lines.

**NOTICE TO CORRESPONDENTS.**

All MS., Letters, Books for Review, and other matters intended for the Editor should be addressed The Editor, The Lodge, Porchester Square, London, W.

The Editor cannot undertake to return rejected MS., even when accompanied by stamped directed envelope.

**"Burdett's Hospital Annual," 1894.**

Fifth year of publication. 700 pp. Boards, gilt lettering. A most complete guide to British, Colonial, Indian, and American Hospitals, Dispensaries, Nursing and Convalescent Institutions, and Asylums. Price 5s. Published by The Scientific Press (Limited), 428, Strand, W.C.

**NOTICE.—The Index for Vol. XIV. (ending Sept. 30th, 1893) is on sale at the Office of this Journal, price 2½d., post free.**

### Medical Vacancies and Appointments.

### APPOINTMENTS.

G. A. Bannatyne, M.D.Glasg., M.R.C.P.Edin., appointed Honorary Physician to the Royal Mineral Water Hospital, Bath. C. A. Lumley, M.R.C.S.Eng., L.R.C.P.Lond., appointed District Surgeon to Kentani, Transkei Cape Colony. F. Montizambert, M.D., appointed General Superintendent of the Canadian Quarantine Service. E. C. Moore, M.B., C.M.Edin., &c., appointed Surgeon to the Edinburgh Ear and Throat Dispensary. F. H. Westmacott, M.R.C.S., L.R.C.P., appointed Resident Medical Officer to the Manchester Royal Infirmary Convalescent Hospital at Cheadle. H. S. Ware, B.A., M.B., B.S.Camb., appointed Resident Medical Officer to the City of London Hospital for Diseases of the Chest, Victoria Park. G. E. Shuttleworth, B.A., M.D., &c., late Medical Superintendent Royal Albert Asylum, Lancaster, appointed Lecturer on Physiology and Hygiene at the Battersea Polytechnic Institute. G. H. Douty, M.A., M.B., B.C., Senior Demonstrator of Anatomy in the University of Cambridge, has been appointed to the Lectureship in Midwifery, vacant by the resignation of Dr. Griffith.

### VACANCIES.

The following vacancies are announced this week, the amount of salary in each case being given after the name of the institution:

### Resident Medical Officers.

Bethlem Hospital. Lambeth Road, S.E.—Two Resident Clinical Assistants. Applications and testimonials, endorsed "Clinical Assistantship," to the Treasurer, Bethlem Hospital, Lambeth Road, S.E., by March 31st.

Birmingham General Dispensary, Birmingham. Resident Surgeon. Salary £150 per annum, with an allowance for cab hire, and furnished rooms, fire, lights, and attendance. Applications and testimonials to Alex. Forrest, Secretary, by April 11th.

Carnarvonshire and Anglesey Infirmary, Bangor.—House-Surgeon. A knowledge of the Welsh language is desirable. Salary, £70 per per annum, with board and lodging in the house. Applications and testimonials to the Secretary by April 7th.

East London Hospital for Children, Glamis Road, Shadwell, E.—House-Physician. No salary, but board, lodging, and washing are provided. Applications and testimonials to Thomas Hayes, Secretary, by April 4th.

Guest Hospital, Dudley. Assistant House-Surgeon. Appointment for six months. No salary, but board, lodging, and washing in the hospital. Applications and testimonials to E. Poole, Secretary, by March 28th.

General Hospital, Birmingham. Two Assistant House-Surgeons. Appointment for six months, and may be held by re-election for a further period of three or six months, but no longer. No salary attached to the posts, but residence, board, and washing will be provided. Applications and testimonials to Howard J. Collins, House Governor.

Metropolitan Hospital, Kingsland Road, N.E.—House-Physician, House-Surgeon, and Assistant House-Surgeon. Appointments tenable for six months. The House-Physician and House-Surgeon will receive a salary of £60 per annum. Applications and testimonials to Charles H. Byers, Secretary, by March 26th.

National Hospital for the Paralysed and Epileptic (Albany Memorial), Queen Square, Bloomsbury.—House-Physician. The present Junior House-Physician is a candidate, and applicants should state whether they are prepared to accept either appointment. The salary of the Senior House-Physician is £100, and of the Junior £50 per annum, with board and apartments. Applications and testimonials to B. Burford Rawlings, Secretary, by March 31st.

West London Hospital, Hammersmith Road, W.—House-Physician and House-Surgeon. Appointments tenable for six months. Board and lodging provided. Applications and testimonials to R. J. Gilbert, Secretary-Superintendent, by March 30th.

### Non-Resident Medical Officers.

County Carlow Infirmary.—Surgeon. Salary £94 per annum. Applications to the Secretary to the Governors by March 20th. Election on April 3rd.

Infirmary for Consumption and Diseases of the Chest and Throat, 26, Margaret Street, Cavendish Square, W.—Honorary Visiting Physician; must reside within one mile of the Institution. Particulars of qualifications to be obtained at the Infirmary.

New Hospital for Women, Euston Road. N.W.—Female Assistant Anæsthetist. Applications and testimonials to Margaret M. Bagster, Secretary, by March 28th.

Owens College, Manchester.—Professor of Zoology. Applications to the Council of the College, under cover to the Registrar, by April 3rd.

Royal College of Physicians.—Milroy Lecturer. Applications to Edward Liveing, M.D., Registrar, by April 9th.

University College, Bristol.—Medical Tutor. Salary, £125 per annum. Applications and testimonials to E. Markham Skerritt, Dean, by April 4th.

West End Hospital for Nervous Diseases, &c., 73, Welbeck Street, W.—Anæsthetist. Appointment for twelve months. Candidate eligible for re-election. Applications to H. Ansell, Secretary.

Yorkshire College, Leeds.—Professor of Pathology. Particulars from the Secretary of the College.

### Books Received.

Messrs. Burns and Oates.

"Life of the Princess Borghese." By Le Chevalier Zeloni. Translated by Lady Martin.

Methuen and Co.

"The Secret of Madame de Monluc." By the Author of "Mademoiselle Mori."

Macmillan and Co.

"Aero-Therapeutics; or, The Treatment of Lung Diseases by Climate.'

"The Lumleian Lectures for 1893." By Charles Theodore Williams, M.D.

Remington and Co.

"Doctor or Lover?" By Faber Vance. 2 vols.

Cassell and Co.

"The Rectum and Anus. Their Diseases and Treatment." By Charles B. Ball. Second edition.

The W. T. Keener Co., Chicago.

"The Technique of Post-mortem Examination." By Ludwig Hektoen, M.D.

"Stricture of the Urethra." By G. Frank Lydston, M.D.

Blackie and Son.

"The Students' Introductory Handbook of Systematic Botany." By Joseph W. Oliver.

Hirschfeld Brothers.

"Food and Drink Rationally Discussed." By Thomas Dutton, M.D.

**Periodicals and Pamphlets.**—The Strand Magazine; the Picture Magazine; Illustrated Penny Tales; the Million; Tit Bits; the Philadelphia Monthly Register; the Johns Hopkins Boulletin; the Chicago Clinical Review; the New York Medical Record; Notes on Bier's New Method of Treating Strumous Diseases of the Extremities by Passive Congestion, by A. G. Miller, F.R.C.S.E.; the Christian World; Izerelmey and Waterproof Walls; the Medical Pioneer; the Charity Organisation Review; the Review of Reviews; the Ladies' Gazette of Fashion; the Utago Witness; Public Health; London; Report of the Port of London Sanitary Committee.

### THE SCIENTIFIC PRESS LIST.

Illustrated, Crown 8vo., 400 pp., nearly ready, price 3s. 6d.,

### HELPS IN SICKNESS AND TO HEALTH.

By HENRY C. BURDETT.

Author of "Hospitals and Asylums of the World," &c. An invaluable work for every household. Second Edition.

"It would be difficult to find one which should be more welcome in a household than this unpretending but most useful book."—*The Times.*

"We can heartily recommend this little epitome of useful information to all who desire to have at hand, in the most accessible form, a ready guide to tell them where to go and what to do, without a moment's doubt or loss of time, where time is so valuable that a few minutes or an hour lost may be irrecoverable in the mischief resulting."—*Spectator.*

Demy 8vo., about 400 pp. [In the Press.

### MEDICAL HISTORY FROM THE EARLIEST TIMES.

By E. T. WITHINGTON, M.A., M.B., Oxon.

This important history of Medicine from the earliest times has been treated in an exhaustive and thorough manner.

It comprises sixty-five chapters and seven appendices, commencing with Medicine in Pre-historic Times, and ending with the Origin of Modern Medicine.

The work, which is in active preparation, will be embellished with some striking copies of ancient inscriptions.

Orders for early copies are being booked, or may be sent through any bookseller.

IMPORTANT NEW HANDBOOK OF MIDWIFERY.

18mo., profusely illustrated with original cuts, tables, &c.; about 260 pp., handsomely bound in leather. Price 6s.,

### A PRACTICAL HANDBOOK OF MIDWIFERY.

By FRANCIS W. N. HAULTAIN, M.D.

This Handbook is produced in a portable and convenient form for reference, and is intended mainly as a practical help to the busy practitioner. It is bound in roan, of a size convenient for the pocket.

To Students it brings forward succinctly the chief points of practical importance in the study of Obstetrics.

"MR. BURDETT'S MONUMENTAL WORK."—*The Times.*

In FOUR VOLUMES: Crown quarto, handsome cloth gilt, bevelled edges, PROFUSELY ILLUSTRATED with PLANS and DESIGNS. Together with a portfolio containing SOME HUNDREDS OF PLANS. Price £8 8s. complete:—

### HOSPITALS AND ASYLUMS OF THE WORLD:

Their Origin, History, Construction, Administration, Management, and Legislation; with Plans of the Chief Hospitals, Asylums, Convalescent Institutions, Special and Infectious Hospitals, Nurses, Homes, and Medical School Buildings, in addition to those of all the Hospitals of London in the Jubilee Year of Queen Victoria's Reign, accurately drawn to a Uniform Scale.

By HENRY C. BURDETT.

Vols. I. and II.—ASYLUMS—HISTORY and ADMINISTRATION, CONSTRUCTION, PLANS, and BIBLIOGRAPHY. Price £4 10s.

Vols. III. and IV.—HOSPITALS—HISTORY and ADMINISTRATION, CONSTRUCTION, PLANS, and BIBLIOGRAPHY. With Portfolio containing some Hundreds of Plans. Price £6.

THE ENTIRE WORK COMPLETE, Price £8 8s.

Of the small edition printed a limited number of copies still remain, and Institutions, Libraries, and Clubs have therefore an opportunity to add this remarkable work to their libraries which cannot much longer continue.

A limited number of Portfolios of Plans will be supplied separately at £3 3s. each.

Foolscap 8vo., cloth gilt, price 2s., post free,

### MYXŒDEMA: And the Effect of Climate on the Disease.

By A. MARIUS WILSON, M.D., B.S., L.R.C.P.Lond., M.R.C.S.Eng.

A valuable little treatise on a subject about which comparatively little has been written. The special feature of this work is its treatment of the effects of climate upon the disease.

Demy 8vo., nearly 200 pp., cloth gilt, profusely illustrated with over 70 special cuts, price 6s., post free,

### ART OF MASSAGE.

By A. CREIGHTON HALE.

A complete text-book of this valuable art. The most practical, simplest, and generally useful work published on this subject. Suitable for self-tuition.

"A volume on the 'Art of Massage,' from the pen of one of its foremost advocates in this country. . . . Admirably illustrated."—*Westminster Review.* "The latest and most complete book we know on Massage."—*Queen.* "A clear exposition of the science. A book to be read by all interested."—*Publishers' Circular.*

Third Edition, About 400 pp., profusely illustrated, crown 8vo. [In the Press.

### COTTAGE HOSPITALS, General, Fever, and Convalescent: Their Progress, Management, and Work.

By HENRY C. BURDETT.

Author of "Hospitals and Asylums of the World," "Pay Hospitals of the World," "Hospitals and the State," "Cottage Hospitals; General, Fever, and Convalescent, with Fifty Beds and Under," "The Relative Mortality of Large and Small Hospitals," "Helps to Health," "Burdett's Hospital Annual and Year Book of Philanthropy," and Editor of THE HOSPITAL.

The demand for copies of this useful and practical work exhausted the Second Edition soon after publication. The Third Edition is revised throughout and brought up to date, and profusely illustrated with new and original drawings.

All interested in the establishment of Cottage Hospitals and small institutions will find this book of practical value, and a reliable authority on all branches of a subject which is beset with so many difficulties.

"Mr. Burdett's work contains all the information which could be required by any one who undertakes to found or to manage a Cottage Hospital, and will, no doubt, be both popular and useful."—*British Medical Journal.*

"The book gives many useful and important particulars relating to Cottage Hospitals."—*Lancet.*

"To all those, and they are now many, who are interested in the work of 'Cottage Hospitals,' Mr. Burdett's compendium on the subject will be found a most useful handbook."—*Standard.*

"Contains a vast amount of practical information, and may on that ground be strongly recommended to all who may wish to found, or may have to manage a Cottage Hospital. They will find, we think, advice founded on experience, on pretty well every subject that they are likely to be called upon to deal with."—*Medical Times and Gazette.*

THE FEEDING OF INVALIDS.

Now Ready. Demy 8vo., cloth gilt, pp. 260. Price 3s. 6d.,

### ART OF FEEDING THE INVALID.

By a MEDICAL PRACTITIONER and a Lady Professor of COOKERY.

A book that the practitioner can place in the hands of the nurse as a guide in his absence.

Matrons of institutions, and all engaged in the care of the sick, have found it invaluable as a daily guide.

This important work fully and clearly describes the nature and tendency of diseases most frequently met with, and gives a general outline upon which the diet of the invalid should be regulated. Corresponding with the chapters are lists of new and original recipes, which are intended for daily use and reference.

The labour and anxiety of providing suitable diet for invalids will, by the use of this book, be reduced to a minimum.

Among the subjects treated are the following: The Feeding of Invalids Generally; Food; Food in Acute Febrile Diseases, in Convalescence and Chronic Illness, in Indigestion, in Gout, in Constipation and Diarrhœa, in Liver and Kidney Diseases, in Diabetes, in Tuberculosis and Consumption, in Corpulence, and for Children.

"The Art of Feeding the Invalid" has the approval of Members of the Faculty, its Medical author being widely known as the author of a standard text-book.

The Recipes are entirely new, having been especially written by a Professional Cook who holds diplomas and has had vast experience as a lecturer.

Now Ready. Demy 8vo., about 150 pp., profusely illustrated with Coloured and other Plates and Drawings. Price 6s. net,

### LECTURES ON GENITO-URINARY DISEASES.

By J. C. OGILVIE WILL, M.D., C.M., F.R.S.E.,

Consulting Surgeon to the Aberdeen Royal Infirmary, and Examiner in Surgery in the University of Aberdeen.

CONTENTS.—Urethral Fever and Catheter Fever—Treatment of Retention of Urine—Gleet and its Treatment—On Varicocele—On Hydrocele—The Treatment of Syphilis—Appendix—Prescriptions for Syphilis, &c., &c., &c.

Now Ready. Crown 8vo, 414 pages, with many handsome plates and portraits, cloth gilt, 2nd edition, rewritten and enlarged, price 2s.,

### PRINCE, PRINCESS, AND PEOPLE.

Being the Public Life of the Prince and Princess of Wales.

By HENRY C. BURDETT.

8vo., 375 pp., 6s. nett, [In the Press.

### CHARITY ORGANIZATION.

A valuable collection of contributions on this important question by leading writers in the United States, Great Britain, and the Continent of Europe. The authors' names comprise:—

| | |
|---|---|
| Rev. F. G. Peabody, | Mr. A. McDougall, |
| Mrs. Chas. R. Lowell, | Mr. R. A. Leach, |
| Miss Frances Morse, | Mr. George Milne, |
| Rev. B. H. Alford, | Mr. H. Willink, |
| Rev. E. H. Bradly, D.D., | Mr. Baldwin Fleming, |
| Rev. Brooke Lambert, | Mr. C. H. Wyatt, |
| Baron von Reitzenstein, | Miss Sharpe, |
| Mr. C. N. Nicholson, | Miss Sturge, |
| Mr. C. P. Larner, | Miss Prideaux, |
| Mr. T. Mackay, | &c., &c. |

London: THE SCIENTIFIC PRESS (LIMITED), 428, STRAND, W.C.

